# Association of CYP24A1 with survival and drug resistance in clinical cancer patients: a meta-analysis

**DOI:** 10.1186/s12885-022-10369-x

**Published:** 2022-12-16

**Authors:** Rui Zeng, Hua Li, Lingyan Jia, Sau Har Lee, Rilei Jiang, Yujia Zhang, Xudong Hu, Tingjie Ye, Xiaoling Wang, Xiaofeng Yan, Yanlin Lu, Zhumei Sun, Jiatuo Xu, Wei Xu

**Affiliations:** 1 School of Basic Medical Science, Shanghai University of Traditional Chinese Medicine, Shanghai, China; 2 ZJU-UoE Institute, Zhejiang University School of Medicine, Zhejiang University, Haining, China; 3 School of Biosciences, Faculty of Health and Medical Sciences, Taylor’s University, Subang Jaya, Lakeside CampusSelangor, Malaysia; 4 Department of Oncology and Institute of Traditional Chinese Medicine in, Oncology, , Longhua Hospital, Shanghai University of Traditional Chinese Medicine, Shanghai, China

**Keywords:** CYP24A1, Survival, Drug resistance, Clinical patients, Meta-analysis

## Abstract

**Background:**

Acquired chemo-drug resistance constantly led to the failure of chemotherapy for malignant cancers, consequently causing cancer relapse. Hence, identifying the biomarker of drug resistance is vital to improve the treatment efficacy in cancer. The clinical prognostic value of CYP24A1 remains inconclusive, hence we aim to evaluate the association between CYP24A1 and the drug resistance in cancer patients through a meta-analysis approach.

**Method:**

Relevant studies detecting the expression or SNP of CYP24A1 in cancer patients up till May 2022 were systematically searched in four common scientific databases including PubMed, EMBASE, Cochrane library and ISI Web of Science. The pooled hazard ratios (HRs) indicating the ratio of hazard rate of survival time between CYP24A1^high^ population vs CYP24A1^low^ population were calculated. The pooled HRs and odds ratios (ORs) with 95% confidence intervals (CIs) were used to explore the association between CYP24A1’s expression or SNP with survival, metastasis, recurrence, and drug resistance in cancer patients.

**Result:**

Fifteen studies were included in the meta-analysis after an initial screening according to the inclusion and exclusion criteria. There was a total of 3784 patients pooled from all the included studies. Results indicated that higher expression or SNP of CYP24A1 was significantly correlated with shorter survival time with pooled HRs (95% CI) of 1.21 (1.12, 1.31), metastasis with pooled ORs (95% CI) of 1.81 (1.11, 2.96), recurrence with pooled ORs (95% CI) of 2.14 (1.45, 3.18) and drug resistance with pooled HRs (95% CI) of 1.42 (1.17, 1.68). In the subgroup analysis, cancer type, treatment, ethnicity, and detection approach for CYP24A1 did not affect the significance of the association between CYP24A1 expression and poor prognosis.

**Conclusion:**

Findings from our meta-analysis demonstrated that CYP24A1’s expression or SNP was correlated with cancer progression and drug resistance. Therefore, CYP24A1 could be a potential molecular marker for cancer resistance.

**Supplementary Information:**

The online version contains supplementary material available at 10.1186/s12885-022-10369-x.

## Background

Drug resistance contributes to the failure of chemotherapy and the subsequent relapse in cancer treatment, eventually causing the death of patients [1–3]. Identification of the resistance biomarker or signaling pathway provides a target to revert the resistant cancer cells to sensitive cells which can then be eliminated by the chemo-drugs effectively. Accumulating evidence reveals that the metabolism of cancer cells is intimately associated with drug resistance, one of the most critical challenges in cancer treatment [4]. From epidemiological studies, vitamin D insufficiency is proven to have an etiological impact in various human cancers [5–7]. Preclinical and clinical studies demonstrated that the metabolites of vitamin D are preventative and are potential therapeutic anticancer agents [8–11].

Vitamin D signaling has been shown to increase sensitivity of cancers to chemo-drugs [12,13]. Consistently, our previous study demonstrated that vitamin D signaling was associated with chemo-resistance of cancer [14]. Physiologically, vitamin D3 is metabolized to 25(OH)D_3_ to produce 1α,25(OH)_2_D_3_, which is also known as calcitriol. Calcitriol is the active form of the hormone that binds to the specific nuclear vitamin D receptor (VDR), which classically regulates gene expression through binding to the DNA promoter [15,16]. The genes activated by calcitriol/VDR binding are normally involved in regulating proliferation, apoptosis, differentiation, and angiogenesis capabilities of cancers [17–20], which are correlated with the resistance of cancer cells. Preclinically, calcitriol has been examined for its therapeutic efficacy in chemo-prevention and anticancer activity [21–23]. The degradation of calcitriol abrogates the VDR signaling and promotes the cancer progression [24].

The mitochondrial inner-membrane cytochrome P450 enzyme, 25-hydroxyvitamin D 24-hydroxylase, encoded by CYP24A1, catalyzes conversion of 25(OH)D_3_ and calcitriol to inactive metabolites [25,26]. Therefore, CYP24A1 plays a role to inhibit the level of calcitriol and the VDR signaling. CYP24A1 was highly unregulated in various cancer types, including lung, colon, and ovarian tumors [27–29]. In several clinical studies, higher expression of CYP24A1 was shown to be correlated with poor prognosis of various cancer types [30–32]. However, there are also other studies suggesting that CYP24A1 had no significant correlation with the overall survival rate among lung cancer patients [33]. Therefore, the association of CYP24A1 expression with the prognosis remains uncertain and needs to be conclusively studied. Additionally, there are also few studies which revealed the polymorphisms of CYP24A1 that were associated with cancer risk and poor prognosis of patients [34,35]. This indicates that the SNP of CYP24A1 might influence the expression or function of CYP24A1, which could potentially impact the cancer occurrence and the prognosis of patients. In our previous study, we had identified CYP24A1 as a potential resistance biomarker for various cancer types. However, the clinical significance of CYP24A1 in prognosis and drug resistance requires further investigation. Hence, a meta-analysis of eligible studies was conducted to determine the association of CYP24A1 expression with the prognosis of cancer patients and the resistance to chemotherapy to clarify the exact prognostic value of CYP24A1 in drug resistance prognosis.

## Materials and methods

### Publication search strategy

Potentially relevant publications were exhaustively searched using a combined medical subheading (MeSH) term in several databases, including PubMed, EMBASE, Cochrane library and ISI Web of Science up to May 2022 with no lower limitation set for the date of publication. MeSH terms related to CYP24A1 (or CYP24*) in combination with words related to cancer (cancer* or adenocarcinoma* or carcinoma* or tumor*), as well as terms related to patient* (or clinic*) were used to retrieve eligible studies.

### Study selection criteria

Studies were screened and selected according to the following criteria: (1) study subject involved human patients; (2) the studies had to measure the CYP24A1 expression level or the SNP which was associated with cancer risk; (3) the studies had to present the overall survival curve data or present the HRs with 95% CIs. Meanwhile, studies were excluded if they met the following criteria: (1) duplicated studies or studies with a repeated analysis; (2) letters, reviews, case reports or conference; (3) study subject involve cell lines or xenografted animals with patient-derived cancer cells; (4) CYP24A1 SNP with unknown function.

### Data extraction

The articles that have fulfilled both inclusion and exclusion criteria were included and reviewed thoroughly by two investigators (X.W. and Z.R.) independently, where vital information were extracted. Any disagreement was discussed and a consensus was reached for all issues. The following information was collected from each study: first author’s name, year of publication, cancer type, ethnicity, the detection approach for CYP24A1, sample size, number of CYP24A1^high/SNP^, number of CYP24A1^low/WT^, treatment, outcome (overall survival), *P* value, HRs (the survival time of CYP24A1^high/SNP^ population vs CYP24A1^low/WT^ population) with 95% CIs from multivariate analysis.

### Quality assessment

The quality of the included studies in meta-analysis was assessed using the Newcastle–Ottawa quality assessment scale (NOS) [36]. The scale includes eight items with three sections: (1) selections [four items, one star for each item], (2) comparability [one item, two stars], and (3) outcome [three items, one star for each item]. Each item was scored after careful evaluation of the studies and the total scores were calculated to quantitatively assess their quality. The highest scores were nine that indicates highest quality while lowest scores were zero that represents lowest quality. Inconsistencies during scoring process from two independent researchers were discussed to reach a consensus agreement.

### Statistical analysis

The pooled hazard ratios (HRs) indicate the ratio of hazard rate of survival time between CYP24A1^high^ population vs CYP24A1^low^ population. The pooled HRs were calculated using HRs with their 95% CIs obtained from the studies in metan package. When the HR data was unavailable in the articles directly, a mathematical estimation based on the survival curve was performed according to the previously published methods demonstrated by Tierney et al. [37–39]. The pooled HRs with 95% CIs were used to evaluate the effect of CYP24A1 expression on the survival time and drug resistance of patients. A pooled HRs > 1 implies that the patients with high expression or SNP of have shorter survival time and are resistant to the drugs. The heterogeneity of included studies in the pooling model was tested using Cochran’s Q test (p_heter_ < 0.05 shows significant heterogeneity) and the I^2^ statistic (I^2^ ≤ 50% shows no or moderate heterogeneity and I^2^ > 50% shows strong heterogeneity). The random-effects model was used in the analysis to avoid significant heterogeneity (p_heter_ < 0.05 and I^2^ > 50%). A sensitivity analysis was conducted to evaluate the stability of the pooling model for pooled HRs by eliminating one study at a time in a sequential manner. The funnel analysis was conducted to estimate publication bias using Begg’s test, with p < 0.05 considered as significant. All statistical analysis was performed using STATA software, version 16.0 (STATA Corporation, College Station, TX, USA). All p values shown were for two-tailed tests.

## Results

### Literature search results

**Fig. 1:**
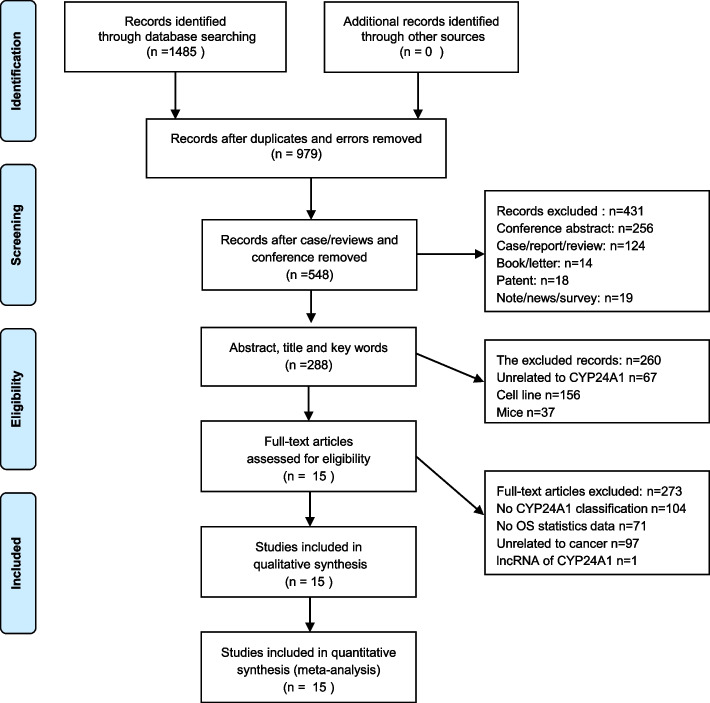
Methodological flow chart for the selection of papers in meta-analysis

A total of 1485 original articles were extracted from the initial search. From the total articles, 506 records were excluded due to duplicated records. 431 records were excluded such as cases/report/reviews, conferences, books/letters, non-relevant records including patents, notes, news and surveys. 260 records were further removed after reviewing the abstract and key words, such as the records unrelated to CYP24A1, the studies using cell lines and mice models. Further refining of studies selection had excluded another 273 records. Finally, 15 studies were selected for meta-analysis. A detailed screening process was illustrated as shown in Fig. 1.

Characteristics and quality assessment of the included studies

The major characteristics of the included studies were tabulated in Table 1. Large number of population samples consisting of 3,784 patients were included from fifteen selected studies. Out of the fifteen studies, the sample size of nine studies is consisted of more than 100 patients. The HRs data of survival time or survival curve of patients was presented in all those studies. Seven out of the fifteen articles studied on lung cancer while the other articles investigated on colorectal cancer (CRC), breast cancer, hepatocellular carcinoma, esophageal cancer, as well as head and neck cancer as shown in Table 1. The patient sample from three out of fifteen articles were from China while the patient sample from another two out of fifteen articles were from North America, as shown in Table 1. CYP24A1 was evaluated at the protein level in four of the studies while four of the studies examined at the mRNA level. Meanwhile, the five studies utilized RT-qPCR to detect the SNP of CYP24A1 which was demonstrated to be associated with cancer risk. Additionally, five of those studies revealed the patients who were under drug treatment. The quality of all the included studies was assessed according to the NOS scale [36,40]. Among those 15 studies, 12 of them scored 8 whereas 3 of them scored 7 as shown in Table 1. The results showed that all the included studies were of high quality.

**Table 1:** Main Characteristics and quality assessment of included 15 studies

Author	Year	Cancer type	ethnicities	CYP24A1 detection	CYP24A1 type	Sample size	CYP24A1^high/SNP^	CYP24A1^low/WT^	Treat ment	Outcome	NOS scale
Chen G ^[[[[30]]]]^	2011	LC	unknown	protein	expr	101	35	66	No	OS	8
Ge N [41]	2017	LC	China	protein	expr	64	31	33	No	OS	8
Sun H [31]	2016	CRC	unknown	protein	expr	99	69	30	No	OS, DFS	8
Porter, R. L [42]	2019	LC	unknown	protein	expr	497	13	485	Chemo	OS	7
Kong, J [33]	2015	LC	China	mRNA	expr	153	na	na	No	OS	8
Borkowski, R [43]	2015	LC	North America	mRNA	expr	182	na	na	No	OS, RFS	8
Mimori, K [44]	2004	EC	Japan	mRNA	expr	42	17	25	No	OS	8
Cai, H [45]	2019	BC	North America	mRNA	expr	1102	646	456	No	OS, RFS	8
Deng, Y. B [46]	2010	HC	unknown	methylation	expr	57	35	22	No	DFS	7
Ramnath, N [47]	2014	LC	unknown	methylation	expr	68	22	46	Chemo	OS, DFS	7
Azad, A. K [48]	2013	HNC	unknown	rs2296241	SNP	522	na	na	Chemo	OS	8
Lancheros, L [49]	2021	LC	Southern Spain	rs6068816	SNP	179	na	na	Chemo	OS, PFS	8
Hlaváč, V [50]	2021	BC	unknown	rs2762934	SNP	369	122	247	Chemo	DFS	8
Gong, C [51]	2017	CRC	northeast China	rs4809957	SNP	264	58	206	No	OS	8
Vidigal, V. M [52]	2017	CRC	unknown	rs6013897	SNP	85	17	68	No	OS	8

Notes: *CRC* Colorectal cancer, *LC* Lung cancer, *EC* Esophageal cancer, *BC* Breast cancer, *HC* Hepatocellular carcinoma, *HNC* Head and neck cancer, *expr* Expression, *Chemo* Chemo-drugs, *OS* Overall survival, *RFS* Relapse free survival, *DFS* Disease free survival

High expression of CYP24A1 was correlated to poor prognosis of cancer patients.

**Fig. 2:**
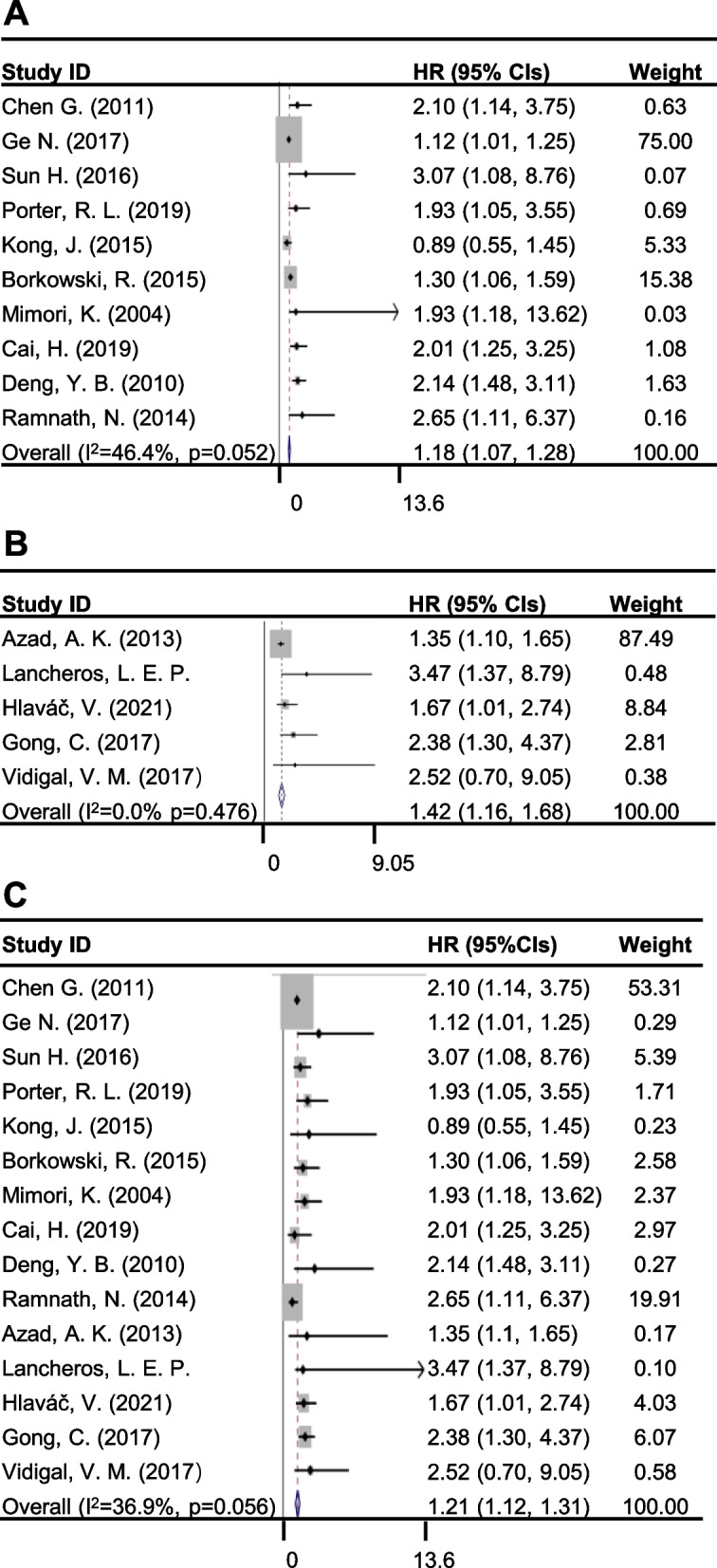
Forest plots of studies evaluating hazard ratios (HRs) for survival in different groups. **A** Forest plots of 10 studies for pooled HRs between CYP24A1^high^ and CYP24A1^low^ population. **B** Forest plots of 5 studies for pooled HRs between CYP24A1^SNP^ and CYP24A1^WT^ population. **C** Forest plots of 15 studies for pooled HRs of overall survival. CI, confidence interval; HR, Hazard ratio

Those 15 selected articles were subjected to multivariate analysis, where random-effects model was used to pool the effect of the CYP24A1 expression on the survival of patients. The pooled HRs (95% CIs) was determined to be 1.18 (1.07, 1.28) in the CYP24A1^high^ population as compared to the CYP24A1^low^ populations where CYP24A1 expression was evaluated at protein level, mRNA level and methylation level (Fig. 2A), indicating that the patients with higher expression of CYP24A1 had a shorter survival time. Afterwards, the HRs (95% CIs) of the populations carrying SNP with certain function in cancer risk were pooled and calculated to be 1.42 (1.16, 1.68) (Fig. 2B), demonstrating that the polymorphisms of CYP24A1 which were correlated to cancer risk were positively associated with poorer prognosis. Finally, the HRs (95% CI) of those 15 studies were pooled and our findings showed that pooled HRs was higher than 1 at 1.21 (1.12, 1.31) (Fig. 2C). This indicates that high expression or SNP of CYP24A1 in cancers led to a shorter survival time. Therefore, these results revealed a significant correlation between CYP24A1 expression and a reduced survival time of cancer patients.

High expression of CYP24A1 was correlated to incidence of metastasis and recurrence of cancer.

**Fig. 3:**
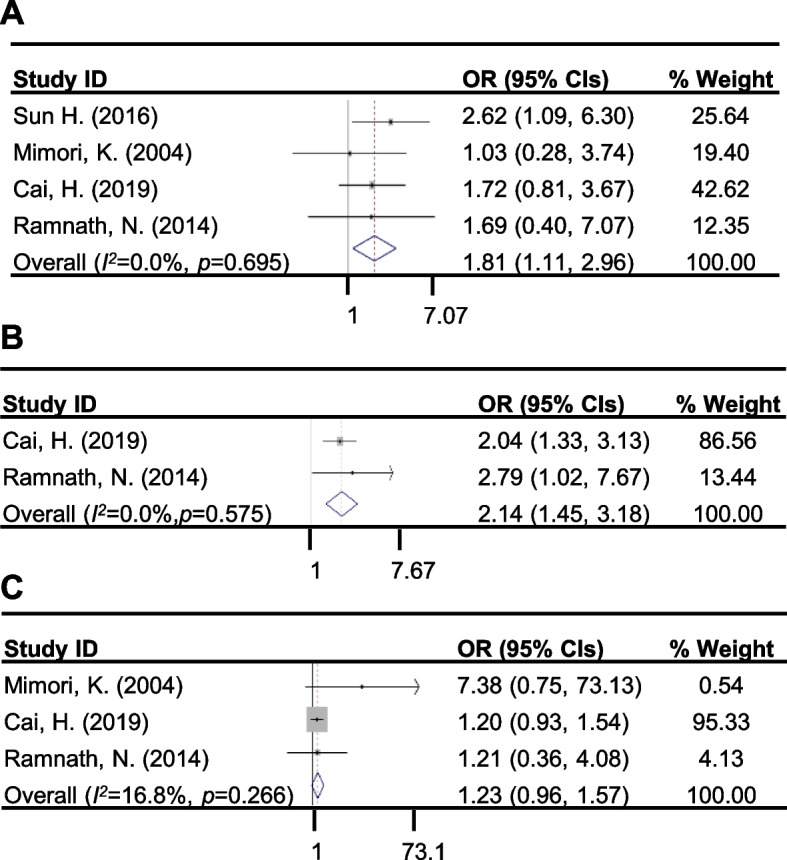
Forest plots of the pooled studies evaluating the association of CYP24A1 expression to metastasis, recurrence and therapeutics. **A** Forest plots of 4 studies for pooled HRs evaluating the association of CYP24A1 expression and metastasis. **B** Forest plots of 2 studies for pooled HRs evaluating the association of CYP24A1 expression with recurrence of patients. **C** Forest plots of 3 studies for pooled HRs evaluating the effect of drug treatment on the expression of CYP24A1. CI, confidence interval; HR, Hazard ratio; OR, odds ratio

Among the CYP24A1^high^ and CYP24A1^low^ populations, the patients included in four of the studies were presented with node metastasis whereas 2 of the studies were identified with recurrence after treatment (Table 2). Since metastasis and recurrence could be correlated to cancer resistance, we calculated and pooled the ORs (odds ratio) of metastasis and recurrence in the CYP24A1 subpopulation to evaluate the effect of CYP24A1 expression on drug resistance. The pooled ORs (95% CIs) were determined to be 1.81 (1.11, 2.96) and 2.14 (1.45, 3.18) for metastasis and recurrence, respectively (Fig. 3A and Fig. 3B). Both pooled ORs and the lower 95% CI values were higher than 1, hence indicating that higher expression of CYP24A1 had indeed promoted the cancer metastasis and recurrence.

**Table 2:** The statistical data of studies

Author	Year	The hazard ratio for survival			The risk ratio of metastasis			The risk ratio of therapeutic			The risk ratio of recurrence		
		HR	lower 95%CI	upper 95%CI	RR	lower 95%CI	upper 95%CI	RR	lower 95%CI	upper 95%CI	RR	lower 95%CI	upper 95%CI
Chen G [30]	2011	2.1	1.14	3.75	N.A	N.A	N.A	N.A	N.A	N.A	N.A	N.A	N.A
Ge N [41]	2017	1.12	1.01	1.25	N.A	N.A	N.A	N.A	N.A	N.A	N.A	N.A	N.A
Sun H [31]	2016	3.07	1.08	8.76	1.32	0.80	2.20	N.A	N.A	N.A	N.A	N.A	N.A
Porter, R. L [42]	2019	1.93	1.05	3.55	N.A	N.A	N.A	N.A	N.A	N.A	N.A	N.A	N.A
Kong, J [33]	2015	0.89	0.55	1.45	N.A	N.A	N.A	N.A	N.A	N.A	N.A	N.A	N.A
Borkowski, R [43]	2015	1.3	1.06	1.59	N.A	N.A	N.A	N.A	N.A	N.A	N.A	N.A	N.A
Mimori, K [44]	2004	1.93	1.18	13.62	1.01	0.55	1.83	4.95	0.60	41.03	N.A	N.A	N.A
Cai, H [45]	2019	2.01	1.25	3.25	1.01	0.91	1.13	1.05	0.92	1.21	1.04	0.94	1.16
Deng, Y. B [46]	2010	2.14	1.48	3.11	N.A	N.A	N.A	N.A	N.A	N.A	N.A	N.A	N.A
Ramnath, N [47]	2014	2.65	1.11	6.37	1.39	0.44	4.39	1.09	0.48	2.45	1.15	0.60	2.19
Azad, A. K [48]	2013	1.35	1.1	1.65	N.A	N.A	N.A	N.A	N.A	N.A	N.A	N.A	N.A
Lancheros, L [49]	2021	3.47	1.37	8.79	N.A	N.A	N.A	N.A	N.A	N.A	N.A	N.A	N.A
Hlaváč, V [50]	2021	1.67	1.01	2.74	N.A	N.A	N.A	N.A	N.A	N.A	N.A	N.A	N.A
Gong, C [51]	2017	2.38	1.3	4.37	N.A	N.A	N.A	N.A	N.A	N.A	N.A	N.A	N.A
Vidigal, V. M [52]	2017	2.52	0.7	9.05	N.A	N.A	N.A	N.A	N.A	N.A	N.A	N.A	N.A

*HR* Hazard Ratio, *CI* Confidence interval, *OR* Odds Ratio

High expression or SNP of CYP24A1 was correlated to drug resistance of cancer patients.

**Fig. 4:**
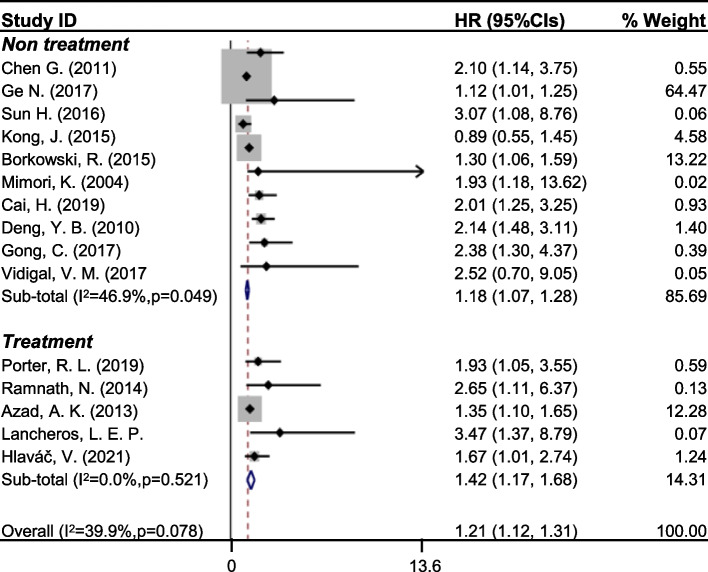
Forest plots of studies evaluating the association between CYP24A1 to prognosis by treatment subgroup analysis. CI, confidence interval; HR, Hazard ratio

To investigate whether drug treatment caused the higher expression of CYP24A1, we had extracted the patients who were under chemotherapeutics among the CYP24A1 groups from 3 different studies (Table 2). The OR was calculated and the pooled ORs was determined to be 1.23 (0.96, 1.57) (Fig. 3C). The revealed data demonstrated that the expression of CYP24A1 in treatment group was higher than the non-treatment group, therefore, suggesting that the drug treatment might promote the increase of CYP24A1 expression. To further confirm that the higher expression of CYP24A1 was actually correlated to drug resistance, we then analyzed the pooled HRs of the survival time in the patients who were treated with drugs in 5 studies and found that the pooled HR (95% CI) was 1.42 (1.17, 1.68) in the treatment subgroup (Fig. 4, Table 3). The results showed that the patients with higher expression or SNP of CYP24A1 had a shorter survival time after treated with drugs, which indicated that elevated expression or SNP of CYP24A1 was highly correlated with the drug resistance.

**Table 3:** Stratified analysis of pooled HRs for cancer patients in different subgroups

Variable	No. of studies	No. of Patients	HR (95% CI)	Heterogeneity			Model
				χ^2^	I^2^	*P Value*	
*Cyp24A1 detection*							
protein	4	761	1.14 (1.02, 1.26)	4.69	36.00%	0.196	random
mRNA	4	1479	1.24 (1.01, 1.46)	4.84	38.10%	0.184	random
methylation	2	125	2.18(1.41, 2.96)	0.13	0.00%	0.717	random
SNP	5	1419	1.42 (1.16, 1.68)	3.51	0.00%	0.476	random
Cancer type							
lung cancer	7	1244	1.15 (1.05, 1.26)	9.03	33.60%	0.172	random
Colorectal cancer	3	448	2.48 (1.13, 3.83)	0.11	0.00%	0.948	random
breast cancer	2	1471	1.82 (1.16, 2.47)	0.168	0.00%	0.614	random
Treatment							
non-treatment	10	2149	1.18 (1.07, 1.28)	16.95	46.90%	0.049	random
treatment	5	1635	1.42 (1.17, 1.68)	3.22	0.00%	0.521	random
Sample size							
> 100	9	3369	1.34(1.17, 1.50)	11.4	0.299	0.18	random
< 100	6	415	1.15(1.03, 1.27)	8.59	0.418	0.127	random
ethnicity							
unknown	8	1798	1.51(1.27, 1.75)	6.53	0.00%	0.48	random
China	3	481	1.11(1.01, 1.23)	3.57	44.00%	0.168	random
North America	2	1284	1.35(1.09, 1.60)	1.81	44.70%	0.179	random

*No*. Number, *HR* Hazard ratio, *CI* Confidence interval

The subsequent question is whether the correlation between CYP24A1 expression and shorter survival time is varied in different cancer types. The analysis revealed that the pooled HRs (95% CI) in lung cancer subgroup was 1.15 (1.05, 1.26), pooled HRs in colorectal cancer subgroup was 2.48 (1.13, 3.83), while pooled HRs in breast cancer subgroup was 1.82 (1.16, 2.47) (Table 3). The pooled HRs for all the 3 subgroups were higher than 1 with lower 95% CI also higher than 1, thus suggesting that CYP24A1 expression is significantly associated with poorer prognosis independent of the cancer type. Moreover, the pooled HRs for the sample size subgroup were also analyzed and both of the pooled HRs were greater than 1 (Table 3), indicating that the sample size did not affect the function of CYP24A1 expression in promoting survival time of cancer patients. Furthermore, the pooled HRs for ethnicity subgroup were analyzed and is shown to be greater than 1, thus indicating that the correlation between higher expression of CYP24A1 with a shorter survival time did not vary with different ethnicities of the included patients. Additionally, the detection approach for CYP24A1 also did not affect the conclusion that patients with high expression of CYP24A1 has a shorter survival time among protein, mRNA and methylation subgroup analysis (Table 3).

### Heterogeneity

The heterogeneity of these 15 studies in the pooled HRs was tested and I^2^ value obtained was 36.9% while p_heter_ is 0.056, which revealed that there was no heterogeneity among these fifteen studies (Fig. 2A). In the CYP24A1 expression subgroup, the I^2^ value was 46.4% and the p_heter_ > 0.05 (Fig. 2B), while the I^2^ value of SNP subgroup was 0.0% with p_heter_ > 0.05 (Fig. 2C). These results indicate that there was no heterogeneity among these studies of CYP24A1 detection subgroup when those studies were pooled (Table 3).

### Sensitivity analysis

**Fig. 5:**
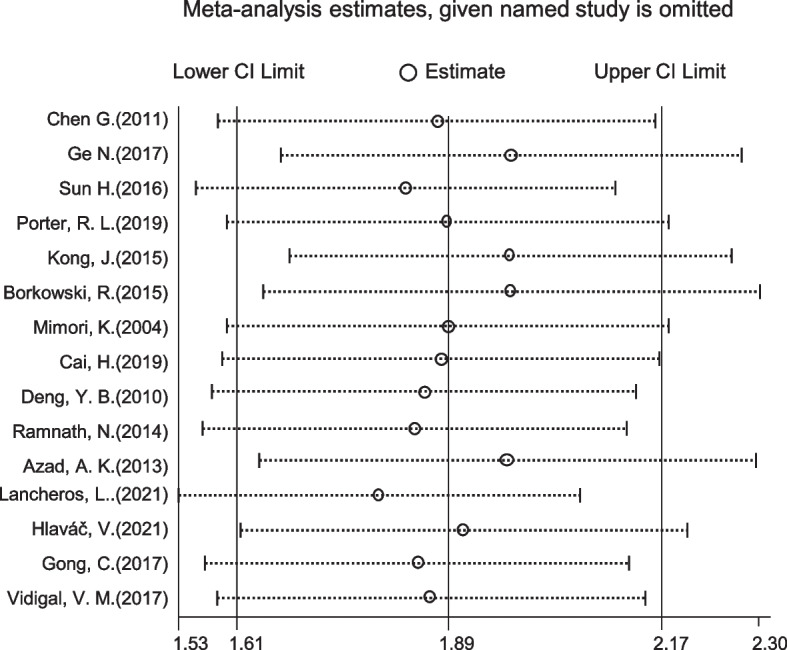
Effect of individual study on the pooled HRs for CYP24A1 and survival. X axis, the ranges of HRs; Y axis, the study ID

TO assess the stability of the analysis model for pooled HRs, the leave-one-out method was applied in the sensitivity analysis. The pooled HRs was sequentially calculated after removing each study to evaluate the effect of removing that study on the pooled results. As shown in Fig. 5, pooled HRs was stable even after each study was removed sequentially, which suggested that any of these studies did not affect the pooled results significantly.

### Publication bias

**Fig. 6:**
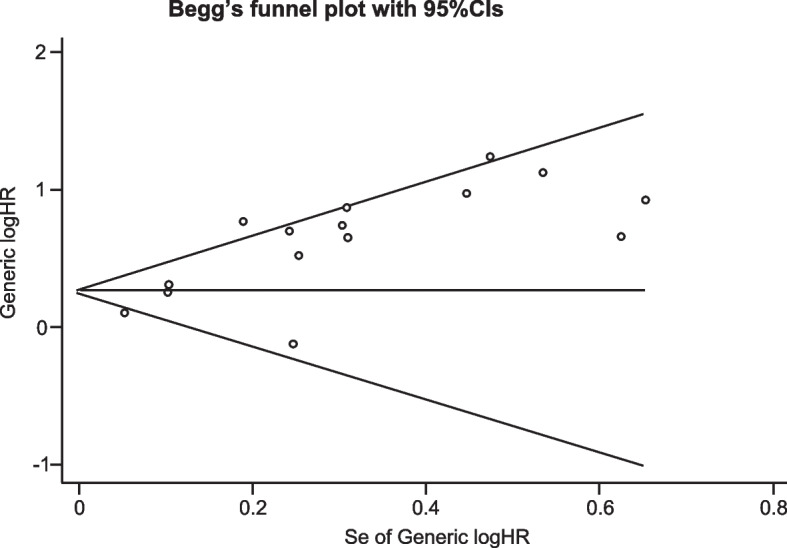
Begg’s funnel plot with 95%CIs for pooled HRs. X axis, the ranges of selogHRs; Y axis, the logHR

Begg’s funnel plot was adopted to evaluate publication bias for all the included studies, where asymmetry was found in the plot (Fig. 6). However, the Begg’s test revealed a p value of 0.216, that is greater than 0.05 (Table 4). Therefore, the presence of a significant publication bias in the meta-analysis cannot be concluded.

**Table 4:** Begg’s test for funnel plot

adj. Kendall's Score (P-Q)	= 25	
Std. Dev. of Score	= 20.21	
Number of Studies	= 15	
z	= 1.24	
Pr > z	= 0.216	
z	= 1.19	(Continuity corrected)
Pr > z	= 0.235	(Continuity corrected)

## Discussion

Calcitriol, the active form of vitamin D metabolites, was proven to have anticancer effect, where intake of vitamin D can significantly reduce the cancer incidence, mortality and improve the survival [53–56]. Besides, calcitriol could also reverse the drug resistance of cancer cells [57–59]. The cellular level of calcitriol was regulated by two dominant enzymes, CYP27B1 and CYP24A1 [60]. CYP27B1 that is highly expressed in kidney, catalyzes the 25(OH)D conversion into an active metabolite, calcitriol, to activate the vitamin D signaling [61,62]. It was reported that breast cancer cells could metastasize to bone tissue more easily when CYP27B1 was knocked-out in mice [63], which indicated that CYP27B1 could potentially suppress the metastasis of cancer cells. On the other hand, CYP24A1 is an enzyme that could degrade the calcitriol to inhibit vitamin D signaling. It was revealed that knocked-down of CYP24A1 gene by siRNA rendered prostate cancer cells to be more sensitive to the growth-suppressive effect of vitamin D3 [64]. Inhibition of CYP24A1 had enhanced the anti-proliferation effect and promoted the activation of caspase-independent apoptosis pathway in prostate cancer cells when exposed to calcitriol [65]. Besides, knocking down CYP24A1 in lung tumor xenograft models had also significantly inhibited the growth of tumors [66]. Therefore, it can be concluded that depletion of CYP24A1 profoundly inhibited the cancer cell proliferation and affected the suppression effect of calcitriol on tumor growth. Moreover, Wang et al. found that knocked-down of CYP24A1 had increased the anti-invasion properties of calcitriol by suppressing the epithelial to mesenchymal transition (EMT) process [67]. When the CYP24A1 was deleted in the Braf^V600E^ tumor mice model, the growth of tumors was attenuated significantly. Contrarily, the overexpression of CYP24A1 in Braf^V600E^ cancer cells had promoted malignant progression and cancer cell resistance to PLX4720 treatment [68]. Taken together, CYP24A1 is involved in the regulation of metastasis and sensitivity to inhibitors in cancer cells, which could be a good candidate for targeted cancer therapy [31,69].

Although numerous studies revealed that CYP24A1 could act as a therapeutic target in cancers and the SNP of CYP24A1 was associated with increased cancer risk and poor prognosis [13,30,49,70,71], it was still unclear whether CYP24A1 expression was significantly related to drug resistance. Therefore, this study innovatively and systematically unveiled the function of CYP24A1 in drug resistance in the clinical patients. Thus far, there have been various studies revealing an association of CYP24A1 polymorphism with survival of cancer patients who are treated with or without drugs. However, some of these studies did not assess the function of CYP24A1 SNP in cancer incidence [72,73], whereas some studies had showed its opposite function in cancer risk and patient survival [74]. Therefore, these cases were excluded whereby only the studies with certain function of SNPs were included in this analysis. Apart from this, a substantial number of studies had showed that the CYP24A1 expression had rendered the cancer cells resistance to drugs in xenograft model [64,75–77]. However, xenografted models and clinical patients possess vast differences, both biologically and disease outcomes. Therefore, the studies involving animal xenografts models were excluded and only the studies with patients who are expressing CYP24A1 were chosen as our study focus. Another side, the studies revealing the methylation of CYP24A1 were included in the analysis due to the methylation was proven to be associated with low expression of CYP24A1 in cancer patients [46,47]. In this meta-analysis that included 15 studies and involved 3,784 patients, the sample size was enough to conduct an analysis to examine the association between CYP24A1 expression with disease prognosis. Findings obtained are conclusive whereby it had demonstrated that CYP24A1 expression was strongly associated with a worse prognosis for OS or RFS in the patients. Interestingly, the patients with higher expression of CYP24A1 possessed a shorter survival time when treated with drugs. Such results indicated that CYP24A1 expression had resulted in poorer prognosis and the heightened drug-resistance in the patients significantly.

Although our findings have proven that CYP24A1 expression is associated with shorter survival and drug resistance, however, there are still some limitations in this study. This is because there may still exist certain degree of bias in this study since it is not possible to eliminate the presence of all potential biases. Firstly, the number of studies included in the meta-analysis was not sufficient. Besides, some of the HRs data were estimated using the strategies reported by Tierney et al. [39], hence the data calculated from the Kaplan–Meier curve may not be as precise as compared to directly obtaining the data from the original article. Moreover, the CYP24A1 expression level was determined through different experimental approaches, such as immunohistochemistry (IHC), RNA-sequencing, and RT-qPCR, thus, the definition of high CYP24A1 may not be consistent. Therefore, the cutoff value of the results may also be varied that could lead to the biasness. Based on the abovementioned factors, a random-effects model was adopted, and subgroup analysis was performed to minimize the impact of these limiting factors.

## Conclusion

This systematic study demonstrated that high expression or SNP of CYP24A1 was positively correlated with shorter survival time in various cancer types. More importantly, the patients who were treated with drugs highly expressed CYP24A1 and had shorter survival time as compared with the subpopulation that expressed lower level of CYP24A1. Such findings suggest that CYP24A1 is associated with the occurrence of drug resistance in clinical patients.Therefore, CYP24A1 could potentially be used as a molecular marker for poor prognosis and cancer resistance.

## Supplementary Information

**Additional file 1.**

## Data Availability

All data generated or analyzed during this study are included in this published article. All methods were carried out in accordance with relevant guidelines and regulations.

## Abbreviations

HR: Hazard ratio
OR: Odds ratio
SNP: Single nucleotide polymorphism
CRC: Colorectal cancer
OS: Overall survival
RFS: Relapse free survival
DFS: Disease free survival
MeSH: Medical subheading
LC: Lung cancer
EC: Esophageal cancer
BC: Breast cancer
HC: Hepatocellular carcinoma
HNC: Head and neck cancer
CI: Confidence intervals
