# Supplementary material for: Association of CYP24A1 with survival and drug resistance in clinical cancer patients: a meta-analysis
Source: BMC Cancer. 2022 Dec 16;22:1317. doi: 10.1186/s12885-022-10369-x (PMC9756477; doi:10.1186/s12885-022-10369-x)
Supplement: Supplementary file 1 — Additional file 1. [file 12885_2022_10369_MOESM1_ESM.docx]

**Search strategy for PubMed database**

1. Click “advanced” button and enter the search page;
2. Input the medical subject headings (Mesh) into the “Add terms to the query box” and then click “or” button;
3. If there are other entry terms for the same Mesh, input the entry terms and choose “or” button to add the terms;
4. Once all the entry terms for the same Mesh were input into the query box, the “search” button can be clicked;
5. Repeat steps 2-4 to input additional Mesh terms;
6. After the search result of all Mesh terms is available, please go to the “history and search details” section in the advanced search page;
7. Click the “three dots” button corresponding to query and choose “Add with AND” command;
8. Once all the search results were added into the query box, click search button.

**Search strategy for Web of Science database**

1. Click “advanced search” button and enter the search page;

2. Input the Mesh term in the “Add terms to the query preview” box and then click the “Add to query” button;

3. If there are other entry terms for the same Mesh, input and choose “or” button before clicking “Add to query” button, then click on the “Add to query” button;

4. Once all the other entry terms for the same Mesh were input into the “Query Preview” box, the “search” button can be clicked;

1. Repeat steps 2-4 to input additional Mesh terms;
2. After all the medical terms were searched, go to the advanced search page and tick the box in the “Session Queries” section;
3. Then click the “Combine sets” and choose “OR”;
4. The combined search result will be loaded automatically.

**Search strategy for Cochrane library database**

1. Click “advanced search” button and enter the search page;
2. Choose “title/abstract/keyword” option before the search box;
3. Input the Mesh terms and other entry terms for the same meaning which were linked by the logical word “or”;
4. Click the “send to search manager” button;
5. Repeat steps 2-4 to input additional Mesh terms;
6. Once all the Mesh terms were sent to the search manager, go to the search manager section;
7. Input the search result in the empty query box by typing “#1”, or other results which were linked with logical word “and”;
8. Then click “continue” button and the result will be loaded automatically.

**Search strategy for Embase database**

1. Click “advanced” button and enter the search page;
2. Input all the terms for the same Mesh term in the search box which were linked with the logical word “or”;
3. Click the “search” button and the search result will be showed on a new page;
4. Repeat steps 2-3 to input additional Mesh terms;
5. Once all the Mesh terms were searched, tick history 1 and history 2 on the history page, then tick the “or” command;
6. Click the “combine” button and the combined search result will be loaded automatically.
